# Ketogenic diet effects on inflammatory allodynia and ongoing pain in rodents

**DOI:** 10.1038/s41598-020-80727-x

**Published:** 2021-01-12

**Authors:** David N. Ruskin, Isabella C. Sturdevant, Livia S. Wyss, Susan A. Masino

**Affiliations:** Neuroscience Program and Department of Psychology, Trinity College, 300 Summit St., Hartford, CT 06106 USA

**Keywords:** Neuroscience, Medical research

## Abstract

Ketogenic diets are very low carbohydrate, high fat, moderate protein diets used to treat medication-resistant epilepsy. Growing evidence suggests that one of the ketogenic diet’s main mechanisms of action is reducing inflammation. Here, we examined the diet’s effects on experimental inflammatory pain in rodent models. Young adult rats and mice were placed on the ketogenic diet or maintained on control diet. After 3–4 weeks on their respective diets, complete Freund’s adjuvant (CFA) was injected in one hindpaw to induce inflammation; the contralateral paw was used as the control. Tactile sensitivity (von Frey) and indicators of spontaneous pain were quantified before and after CFA injection. Ketogenic diet treatment significantly reduced tactile allodynia in both rats and mice, though with a species-specific time course. There was a strong trend to reduced spontaneous pain in rats but not mice. These data suggest that ketogenic diets or other ketogenic treatments might be useful treatments for conditions involving inflammatory pain.

## Introduction

Acute inflammation is a process in which the innate immune system reacts to tissue infection, irritation, or damage to destroy the infecting pathogen, remove the irritating agent and begin repair of tissue damage. As such, it is a normal and beneficial process. If inflammation is not resolved, however, it will become a chronic state in which healthy tissue is harmed. Chronic inflammation is intertwined inexorably with chronic oxidative stress and elevated levels of free radicals and reactive oxygen species^1^. Oxidative stress is a key player in many chronic inflammation-related dysfunctions peripherally^2–5^ and centrally^6–9^. Notably, pain is a major symptom in many inflammation-related disorders, including diabetic neuropathy, chemotherapeutic neuropathy, gout, rheumatoid arthritis, inflammatory bowel disease, and fibromyalgia. Often the pain takes the form of allodynia, a state in which innocuous stimuli are perceived as painful.

Prior studies have shown that diet can influence inflammatory pain, e.g.^10–12^. There is growing evidence that the ketogenic diet (KD) is anti-inflammatory. This diet was introduced as an anticonvulsant treatment for drug-refractory epilepsy, and has very low carbohydrate content as a means to induce a metabolic state similar to fasting but without caloric restriction. In this metabolic state, the liver converts fatty acids to ketone bodies which circulate to be used by other tissues as fuel during reduced availability of glucose; a ketone body-based metabolism should produce fewer free radicals than one based on glucose^13^. The KD should reduce inflammation as it enhances various antioxidant mechanisms^14–26^. Indeed, KD treatment reduces reactive oxygen species^17,18,27–32^, limits oxidative damage to DNA^17,20,33^, lipids^29,34–37^, and proteins^16,20,23,35^, and modulates immune cell function^28,38^. Most of these studies did not use a calorie-restricted KD. In spite of the conceptual overlap among inflammation, pain, oxidative stress, and the KD, there has been very little work on the KD as a treatment for inflammatory pain. Here we tested a KD in a rodent model of experimental inflammatory pain, specifically investigating allodynia and measures of spontaneous pain.

## Methods

All procedures were performed in accordance with the NIH Guide for the Care and Use of Laboratory Animals, and approved by the Institutional Animal Care and Use Committee of Trinity College (A3869-01). Male Sprague–Dawley rats and male C57Bl/6 mice were bred in-house (original stock from Charles River), and pair-housed (rats) or group-housed (mice) in 12 h:12 h light:dark conditions. Treatment started at 10–16 week of age (rats) or 6–8 week (mice). Animals remained on their control diet (CD; LabDiet 5001) or were switched to a KD (BioServ 3666); all diets were provided ad libitum. KD was replaced daily. Diet treatment proceeded for three or four weeks before behavioral studies began, and continued through the end of experimentation. Animals were gently handled daily for several days before testing, to reduce handling stress during behavioral studies and to make animals docile for paw volume testing. All testing occurred during the lights-on period of the daily cycle. For rats, 100 µl complete Freund’s adjuvant (CFA; Thermo Scientific, 0.5 mg/ml) was injected intraplantar into the right hindpaw with the needle tip as close to the center of the footpads as possible; for mice, injections were similar apart from reducing the dose of CFA to 20 μl. All CFA injections were performed in the morning to keep the four h time point well within the light cycle. Volumes of rat right hindpaws before CFA injection did not differ between CD- and KD-fed groups (p > 0.50), such that the 100 µl dose of CFA can be considered equivalent in the two groups.

**Figure 1:**
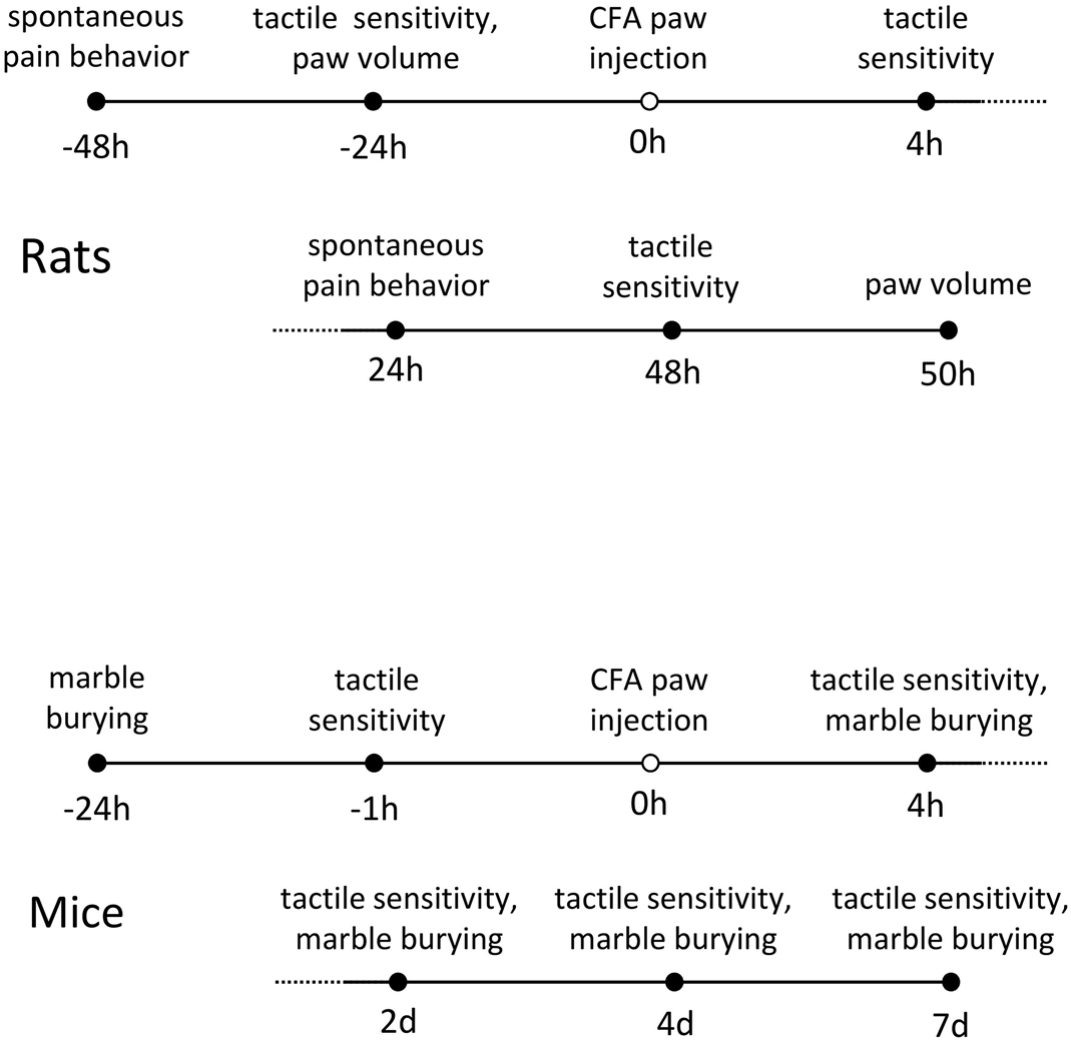
Timeline of experimental procedures in rats and mice.

Before tactile sensitivity testing, rats were habituated by being brought in their home cage into the testing room for 15 min, then placed on an elevated mesh stand in 20 × 20 × 12 cm acrylic enclosures (to minimize locomotion; IITC) for another 15 min; mice were habituated by being placed on the mesh stand in 10 × 10 × 12 cm acrylic enclosures (IITC) for 60 min. At various times before and after CFA injection, tactile sensitivity was measured with an electronic von Frey probe (IITC, Fig. 1). The rigid von Frey probe was applied alternately to each hindpaw until the animal either withdrew the paw or allowed it to be lifted by the probe; the maximum force on each trial was recorded. Three trials with no less than a 120 s intertrial interval occurred per hindpaw. If the animal began to take a step or otherwise shift its position during a trial, that trial was repeated.

In rats, spontaneous pain behavior was assessed 48 h prior to and 24 h after CFA injection. After a 15 min habituation to the testing room, animals were placed singly in 19 × 29 × 13 cm clear-bottom cages for 30 min and filmed vertically from below. Later, videos were watched for indications of spontaneous pain for 60 s every five minutes; videos were watched by two scorers who were independent and blind to dietary treatment and pre- versus post-CFA state. Epochs with grooming or locomotion were avoided. Position of each hindpaw was placed into the following categories: 0, normal weight bearing; 1, light weight bearing; 2, only paw edge touching floor; 3, paw nearly raised off floor; 4, paw completely raised; 5, licking raised paw, and time spent in each position was recorded. For each observed minute, a weighted pain score was calculated (t_1_ + 2t_2_ + … 5t_5_, in which t_x_ is the time spent in category x) and averaged across the six observed minutes^39^. This test was not used in mice as preliminary experiments indicated that mouse paws were too small to reliably distinguish the categories.

In rodents, ongoing painful states interfere with ongoing behavior^40–42^. We assessed this effect in mice with marble burying^43–45^ at the indicated times (Fig. 1). Animals were habituated to the testing room for 30 min, then placed individually in 19 × 29 × 13 cm cages for 30 min. These cages had a 3 × 5 array of 1.6 cm diameter black marbles placed on five cm of wood ship bedding. Photos were taken vertically from above before and after the session and analyzed with Photoshop. The quick selection tool was used to measure the total number of pixels associated with the black marbles, and the polygonal lasso tool was used to measure the number of pixels of the bedding field; the number of marble pixels was expressed as a percentage of the entire field’s pixels. This calculation was performed on before and after pictures, with the comparative decrease in the marble percentage compared to before indicating the amount of burying. Data from two mice that demonstrated very little burying in the baseline test were excluded from analysis. The marble burying test was not used in rats as preliminary experiments indicated that rats did virtually no burying under our conditions.

Volumes of rat hindpaws were measured by water volume displacement in 25 ml graduated cylinders 24 h prior to and 50 h after CFA injection. At sacrifice of subjects, glucose and the ketone body β-hydroxybutyrate were measured in tail vein blood with Precision Xtra meters (Abbott).

T-tests were used for CD versus KD comparisons, with significance indicated by pound signs. For comparisons of post-CFA time points to baseline time points, multiple Bonferroni comparisons were made, with significance indicated by asterisks. Comparisons were considered significant if p < 0.05. All data are presented as mean ± standard error.

## Results

**Figure 2:**
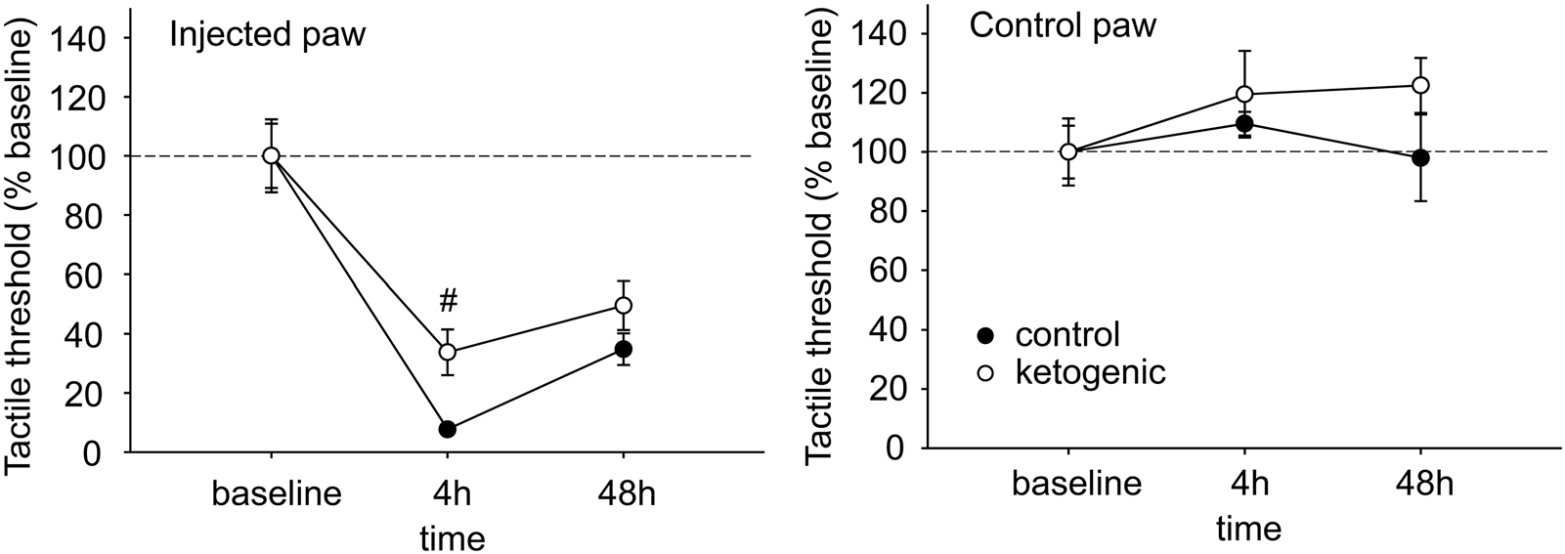
Effects of CFA and diet treatment on tactile allodynia in rats assessed by electronic von Frey probe. For the injected paw, all 4 h and 48 h groups are significantly different from baseline (not indicated). There were no treatment effects on control paw sensitivity. Control n = 14, ketogenic n = 12. ^#^p < 0.05 control v. ketogenic diet.

Plantar tactile sensitivity was low in baseline and not different between CD- and KD-fed rats in injected paws (CD 85.0 ± 7.0 g, KD 85.4 ± 10.6 g, p > 0.50) or uninjected paws (CD 95.6 ± 7.3 g, KD 80.8 ± 9.2 g, g > 0.20). All rats demonstrated strong allodynia of the injected hindpaw; however the magnitude of this effect was significantly smaller at 4 h post-injection in KD-fed animals (Fig. 2). This difference did not remain significant at the 48 h time point (Fig. 2). As expected, plantar tactile sensitivity did not change with diet or time in the uninjected hindpaw (Fig. 2).

**Figure 3:**
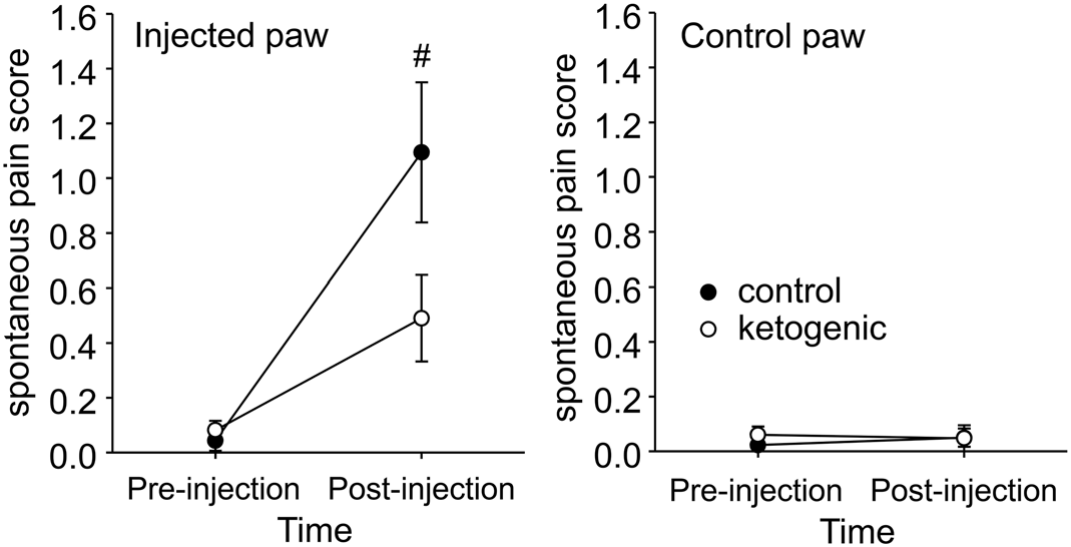
Effects of CFA and diet treatment on spontaneous expression of pain in rats. The spontaneous pain score was calculated from observations on paw favoring, lifting, and licking before and 24 h after CFA injection. For the injected paw, spontaneous pain was significantly higher compared to pre-injection for both groups (not indicated). However, there was a strong trend for spontaneous pain to be lower in ketogenic diet-treated rats. There were no effects regarding the uninjected paw. Control n = 14, ketogenic n = 12. ^#^p = 0.059.

Behaviors indicative of ongoing pain states related to the hindpaws such as licking and avoiding weight bearing were essentially absent pre-CFA, as expected (Fig. 3). After injection, such behaviors were present and directed to only the injected hindpaw in all rats; however there was a strong trend toward less such behavior in KD-fed rats (Fig. 3).

**Figure 4:**
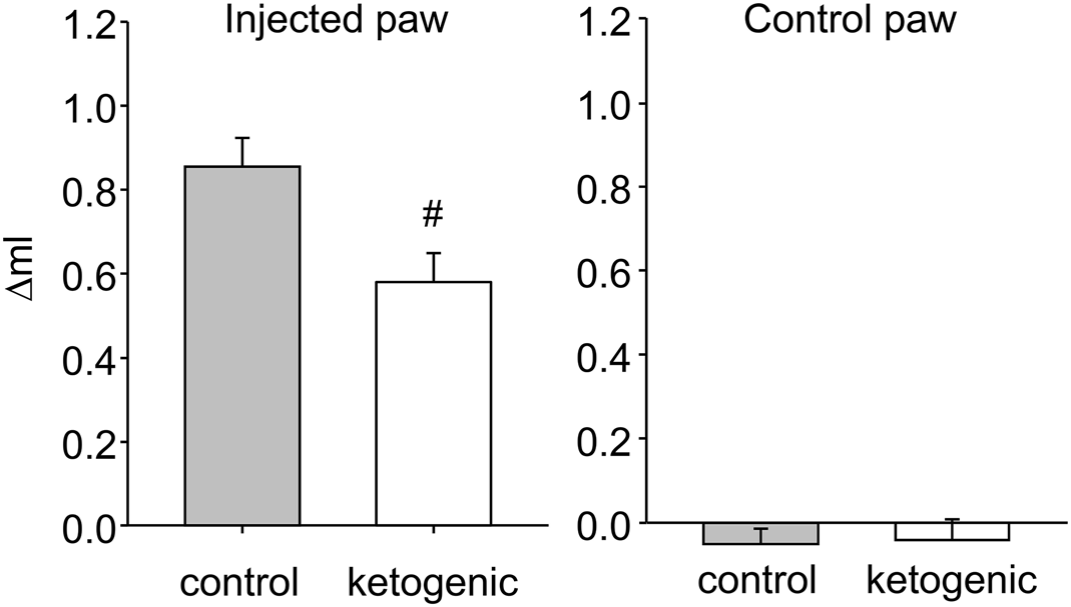
Effects of dietary treatment on CFA-induced inflammatory paw swelling in rats. Paw volume was expressed as the difference between volume at 50 h after CFA injection and volume at baseline. There was significantly less swelling in rats fed the ketogenic diet. There was no effect in control paws. Control n = 14, ketogenic n = 10. ^#^p < 0.05.

As expected^46^, CFA-induced hindpaw swelling was significantly reduced by KD treatment, measured by change in volume from baseline (Fig. 4). The uninjected hindpaw was unaffected (Fig. 4).

**Figure 5:**
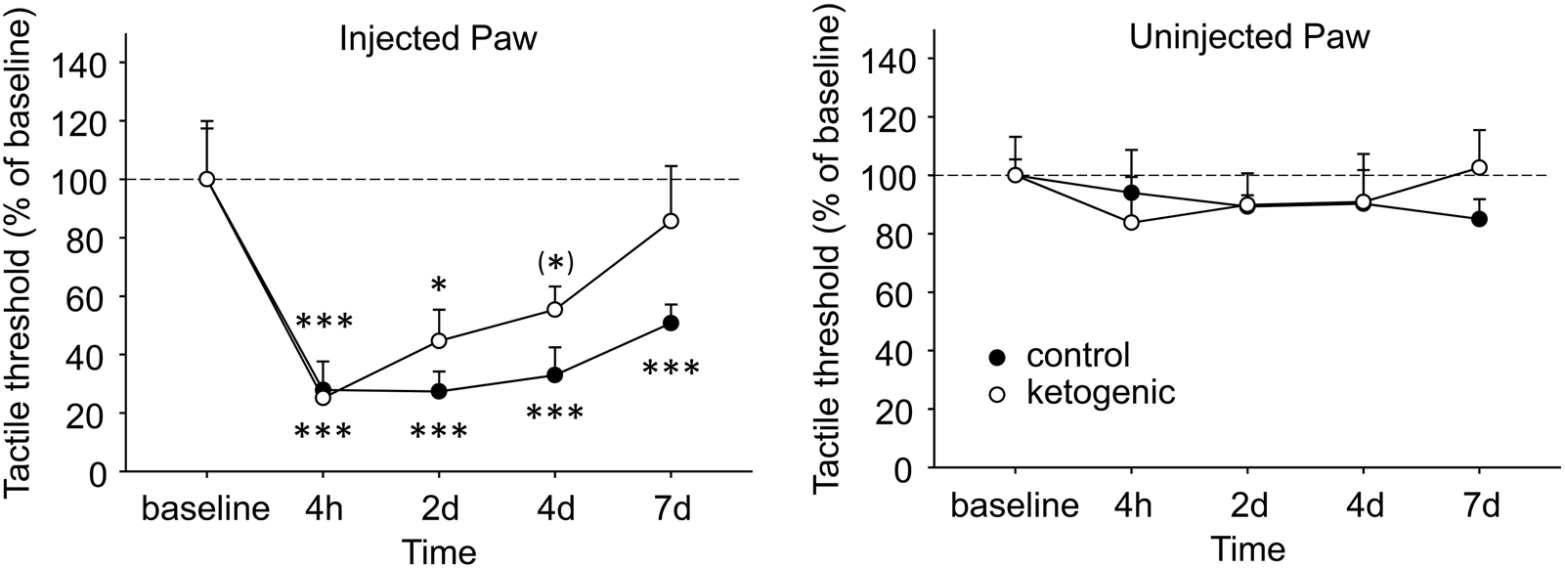
Effects of CFA and diet treatment on tactile allodynia in mouse assessed by electronic von Frey probe. Injected paws became hypersensitive after CFA injection, but the rate of recovery differed in the groups. Control diet-fed mice were still strongly hypersensitive at the last examined timepoint, whereas a gradual and complete recovery occurred in ketogenic diet-fed mice. There were no effects in the uninjected paw. Control n = 6, ketogenic n = 8. ***p < 0.001, **p < 0.05, ^(^*^)^p = 0.071 compared to baseline.

Plantar tactile sensitivity was low in baseline and not different between CD- and KD-fed mice in injected paws (CD 3.18 ± 0.63 g, KD 2.72 ± 0.47 g, p > 0.50) or uninjected paws (CD 2.84 ± 0.15 g, KD 2.46 ± 0.32 g, g > 0.30).). All mice demonstrated robust tactile allodynia of the CFA-injected hindpaw; however, in CD-fed mice significant allodynia remained out to the last time point assessed (7d), whereas in KD-fed mice allodynia was starting to reverse at 2d and tactile sensitivity was no longer different from baseline at 4d (Fig. 5). As expected, plantar tactile sensitivity did not change with diet or time in the uninjected hindpaw (Fig. 5).

**Figure 6:**
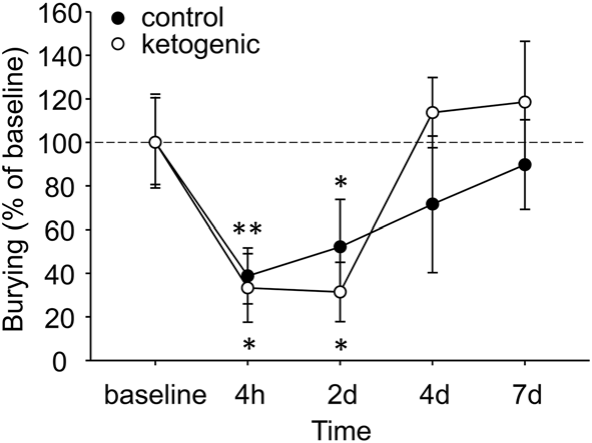
Effects of ongoing pain and dietary treatment on marble burying in mice. CFA injection reduced marble burying in all mice, with no effect of dietary treatment. Control n = 5, ketogenic n = 7. **p < 0.01, *p < 0.05 compared to baseline.

Baseline marble burying was not different between CD- and KD-fed mice (CD 45.2 ± 9.5% buried, KD 32.3 ± 4.7% buried, p > 0.20). Marble burying in mice was strikingly reduced after CFA injection, likely indicating an ongoing pain state (Fig. 6). Burying behavior slowly recovered in both diet groups; and statistics indicated that there was no diet-related difference in recovery rate. Notably, at 4 and 7 days the KD group is burying at levels above baseline, albeit non-significantly, something not found in the CD group (Fig. 6).

Ketogenic diet treatment strongly elevated blood ketone bodies in both species, reduced blood glucose in mice, and produced a trend for decreased mouse body mass (Table 1).

**Table 1:** Effect of diet treatment on physiological parameters.

Species	Diet	β-hydroxybutyrate (mM)	Glucose (mg/dL)	Body mass (g)
Rat	Control	0.25 ± 0.06	129.4 ± 4.3	415 ± 58
	Ketogenic	1.14 ± 0.14***	133.6 ± 9.7	418 ± 50
Mouse	Control	0.42 ± 0.05	170.5 ± 9.2	24.1 ± 0.5
	Ketogenic	2.78 ± 0.30***	99.2 ± 11.0**	20.2 ± 1.6^(^*^)^

Physiological measures were taken after the end of behavioral testing. Control v. ketogenic ***p < 0.001, **p < 0.01, ^(^*^)^p = 0.06. Rat: n = 12 CD, 14 KD; mouse: n = 6 CD, 8 KD.

## Discussion

We found that treatment with a KD significantly ameliorated CFA-induced tactile allodynia in two model species, with more modest effects on indices of ongoing spontaneous pain. A KD-induced amelioration of induced inflammatory pain in rodents is consonant with the KD’s reduction of inflammation itself produced by various insults in various tissues in rodents^19,28,31,46–53^ and patients^54–56^, and reducing pro-inflammatory cytokines^19,28,37,47,49,57–60^ and eicosanoids^55,58^ while elevating anti-inflammatory cytokines^60,61^. These results may appear to contradict a large body of literature showing that high-fat diets promote inflammation^62^. However, this literature refers to diets such as the Western or standard American diet (SAD), high in fat but not low in carbohydrates. The metabolic response to dietary fats differs greatly depending on the presence of carbohydrates: the high‐fat‐plus‐carbohydrate diet promotes fat storage, whereas the high fat, low‐carbohydrate diet promotes fat metabolism^63^. Recently, KD feeding was shown to reverse the tactile allodynia produced in a mouse model of metabolic syndrome^64^ (strikingly, the KD in this study had almost twice the fat level of the high-fat/moderate carbohydrate diet that induced metabolic syndrome). Metabolic syndrome-related allodynia relates to inflammation in the peripheral nervous system^65^, and as diabetic neuropathy is thought also to involve inflammation in the spinal cord^66^, beneficial effects of the KD against pain syndromes involving inflammation could be peripheral, central, or both. KD treatment, however, appears not to be equally effective in all neuropathic pain syndromes^67^ possibly relating to involvement of inflammation*.*

Clinical work with KD and pain had a very early start, specifically regarding migraine^68,69^ and this use may be undergoing a resurgence^70^. Notably, oxidative stress has been hypothesized to be the trigger of many types of migraines^71^. KD feeding effectively treats pain and other symptoms in inflammatory bowel syndrome^72^ and Parkinson’s disease^73^. Overall body pain is alleviated in overweight diabetic patients^74^, although it was not specified if the type of pain was neuropathic. Given the metabolic parallels between KD treatment and fasting, and the established efficacy of fasting against rheumatoid arthritis^75,76^, a KD could be particularly useful in this disorder. Existing studies suggest little clinical benefit^77,78^; however, KD treatment in these studies lasted only seven days (to parallel a fasting treatment, which was itself effective). We have found that antinociceptive effects of the KD evolve over days to weeks^79^ and others have found a similar pattern in reduced oxidative stress^18^, suggesting that longer treatment durations should be attempted in rheumatoid arthritis.

There were several differences between the results with rats and mice. Mice appeared to be more affected physiologically by ketogenic diet treatment, with lowered glucose and a trend to lower body mass (not occurring in rats) and a more than two-fold higher elevation in β-hydroxybutyrate than in rats. These are clearly species related. There were also differences in behavioral outcomes. The ketogenic diet improved tactile allodynia in rats at four hour post-injection, but not later, whereas beneficial effects on tactile allodynia in mice appeared in the two to four day range and persisted thereafter. Given relative species paw sizes and the currently used doses of CFA, the effective dose in mice is substantially higher, and so either or both dose and species could underlie these differences in diet responsiveness. Ongoing pain states were unaffected by diet in mice, with a trend to improvement in rats. Species differences, however, led us to use different tests for each species. The mouse marble burying test clearly showed that a state of ongoing pain reduced performance of this behavior. The lack of a ketogenic diet effect indicates that either that the diet does not improve spontaneous pain in this model in mice, or that marble burying is inappropriate behavior to assess these types of changes.

It is somewhat unclear which of the main metabolic actions of a KD (elevated ketone bodies, lowered and less variable glucose) leads to limiting inflammation and inflammatory pain. Certainly, chronically elevated glucose is undesirable: high fasting glucose and/or impaired glucose tolerance associate with elevated blood cytokines^80–85^, C-reactive protein^80–82,86^, oxidative markers^87^, circulating white blood cells^88^, and inflammatory response of white blood cells^89^. In fact, acute hyperglycemia elevates circulating cytokines through an oxidative mechanism^90^. On the other hand, elevated ketone bodies themselves seem to have beneficial effects: in vivo and in vitro studies show that β-hydroxybutyrate itself moderates the endoplasmic reticulum stress-induced inflammasome^91,92^ and the NRLP3 inflammasome^49,60,92–94^ in various organs in a manner apparently unrelated to its use as a substrate for the tricarboxylic acid cycle^93^. In addition, free fatty acids from a KD might be directly beneficial by reducing mitochondrial production of reactive oxygen species^27^. Besides being anti-inflammatory, there are other possible mechanisms for a KD to limit pain^95^. Regardless of mechanism, this study and a growing body of evidence suggest that pain be added as a variable in more clinical studies of the KD generally, and specifically that more studies of KD treatment in clinical inflammatory pain syndromes is warranted.
